# Identification of the anti-breast cancer targets of triterpenoids in Liquidambaris Fructus and the hints for its traditional applications

**DOI:** 10.1186/s12906-020-03143-8

**Published:** 2020-11-27

**Authors:** Ping Qian, Xiao-Ting Mu, Bing Su, Lu Gao, Dong-Fang Zhang

**Affiliations:** Department of Pharmacognosy, School of Pharmacy, China Medical University, Shenyang, 110122 China

**Keywords:** Liquidambaris Fructus, Breast cancer, Triterpenoids, Network pharmacology, Molecular docking, ErbB4, EGFR

## Abstract

**Background:**

Liquidambaris Fructus is the infructescences of *Liquidambar formosana* Hance and it has been used to treat some breast disease in Traditional Chinese Medicine. In the previous study we found the anti-breast cancer effect of triterpenoid in Liquidambaris Fructus. This study is a further investigation of the triterpenoids in Liquidambaris Fructus and aims to identify their anti-breast cancer targets, meanwhile, to estimate the rationality of the traditional applications of Liquidambaris Fructus.

**Methods:**

Triterpenoids in Liquidambaris Fructus were isolated and their structures were identified by NMR spectrums. Potential targets of these triterpenoids were predicted using a reverse pharmacophore mapping strategy. Associations between these targets and the therapeutic targets of breast cancer were analyzed by constructing protein-protein interaction network, and targets played important roles in the network were identified using Molecular Complex Detection method. Binding affinity between the targets and triterpenoids was studied using molecular docking method. Gene ontology enrichment analysis was conducted to reveal the biological process and signaling pathways that the identified targets were involved in.

**Results:**

Thirteen triterpenoids were identified and 6 of them were the first time isolated from Liquidambaris Fructus. Predicted ADME properties revealed a good druggability of these triterpenoids. We identified 18 protein targets which were closely related to breast cancer progression, especially triple-negative, basal-like or advanced stage breast cancers. The triterpenoids could bind with these targets as their inhibitors: hydrophobic skeleton is a favorable factor for them to stabilize at binding site and polar C17- or C3- substituent was necessary for binding. GO enrichment analysis indicated that inhibition of protein tyrosine kinases autophosphorylation might be the primary mechanism for the anti-breast cancer effect of the triterpenoids, and ErbB4 and EGFR were the most relevant targets.

**Conclusions:**

The study revealed that triterpenoids from Liquidambaris Fructus might exert anti-breast cancer effect by directly inhibit multiple protein targets and signaling pathways, especially ErbB4 and EGFR and related pathways. This study also brings up another hint that the traditional applications of Liquidambaris Fructus on hypogalactia should be reassessed systematically because it might suppress rather than promote lactation by inhibiting the activity of ErbB4.

**Supplementary Information:**

The online version contains supplementary material available at 10.1186/s12906-020-03143-8.

## Background

Liquidambaris Fructus (LF), also called Lu Lu Tong, is the dry infructescences of *Liquidambar formosana* Hance. LF is believed to unclog the meridians (Jingluo) in Traditional Chinese Medicine, and because it mainly affect the liver meridian which is considered closely related to mammary function, therefore LF has been used to treat some breast disease such as hypogalactia [1], mastitis [2], mammary duct ectasia [3], mammary gland hyperplasia [4], etc. Breast cancer is the severest breast disease threatened women’s health as its high incidence rates and mortality rates [5]. Many natural products, including Chinese herbal medicines, have been used in breast cancer treatment, and pharmacological evidence has been accumulated for their application. In the previous research we found that betulonic acid, a triterpenoid come from LF, could inhibit human breast cancer MCF-7 cells proliferation, and induce the cell cycle arrest and apoptosis [6]. Hence it is necessary to further investigate the chemical ingredients and the mechanism of anti-breast cancer effect of LF.

Therefore, in the present study, 13 triterpenoids were identified and 6 of them were the first time isolated from LF. Considering that vast majority of the compounds reported in LF were triterpenoids [7–12], investigating macromolecular targets of LF triterpenoids could provide valuable information. Accordingly we adopted PharmMapper server as the computational tool which utilize a large-scale reverse pharmacophore mapping strategy [13]. On the other hand, we also collected the known and explored therapeutic protein targets of breast cancer. Because proteins do not function in isolation, and proteins which interact with the disease proteins may also play an important role in disease progression [14], thus we constructed protein-protein interaction (PPI) network to display associations between the predict targets of LF triterpenoids and the therapeutic targets of breast cancer. However, this guilt-by-association method generated a large PPI data set that needs further analysis to provide more effectively information [15]. Because cellular processes are mainly carried out by protein complexes [16], we identified the protein complexes from the PPI network using Molecular Complex Detection (MCODE) method, a graph theoretic clustering algorithm that detects densely connected regions that may represent molecular complexes [15, 17]. Molecular docking method could be used to analyses the conformations and orientations of the ligand binding to protein receptor, and predict molecular interactions and calculate binding energies [18], thus it was adopted to identify the targets that had high binding affinity to LF triterpenoids. Gene ontology (GO) enrichment analysis was finally conducted to reveal the biological process and signaling pathways that the identified targets were involved in. By means of the described methodology, this research could help us to acquire a better understanding about the anti-breast cancer potential and the traditional applications of LF.

## Methods

### Chemical ingredients investigation

Open column chromatography (CC) separation was carried out using silica gel (200–300 mesh) and Sephadex LH-20 as stationary phase. NMR spectra were run on a Bruker AVANCE II 600 spectrometer (600 MHz for 1H NMR and 150 MHz for 13C NMR) with trimethylsilane as an internal standard. LF was collected in Jiangsu Province of China in February, 2014, and was identified by one of the authors, Prof. Dong-Fang Zhang. Voucher specimens have been deposited in the Department of pharmacognosy, School of Pharmacy, China Medical University (Liaoning, China).

LF (9 kg) was grinded and refluxed with 70% ethanol, and then concentrated under reduced pressure to afford a residue 430 g. The residue was dispersed in water and successively extracted with cyclohexane and EtOAc, and 13 compounds were isolated from the EtOAc extraction (separation process was shown in Additional file 1). These compounds were determined using ^1^H-NMR and ^13^C-NMR spectrum analysis, and were identified by comparing the results with literature [7–12, 19–24, 75, 76].

### Targets exploration

Absorption and metabolism properties of LF triterpenoids were predicted using admetSAR (http://lmmd.ecust.edu.cn/admetsar1) [23]. Potential targets of LF triterpenoids were predicted using PharmMapper server (http://www.lilab-ecust.cn/pharmmapper/) with the target set Human Protein Targets Only (v2010, 2241) [13]. The known and explored therapeutic targets of breast cancer (ICD10: C50) were collected from the Therapeutic Target Database (http://bidd.nus.edu.sg/group/cjttd/) [24]. Target gene names were normalized using the Uniprot database (https://www.uniprot.org/) [25]. PPI network was constructed using the STRING database (version 10.5, https://string-db.org/) [26], and the network data was processed using Cytoscape software (version 3.6.1) [27] and the isolated nodes were removed. Three parameters, Degree, Closeness Centrality and Betweenness Centrality were used to assess the topological properties of the nodes in the PPI network, and higher values means more topological importance. The nodes whose values were less than the medians were defined as non-critical nodes and were removed from the PPI network. Then the network was analyzed using MCODE (version 1.5.1) [15], and the values of Degree cutoff, Node Score Cutoff, K-Core, Max Depth were 2, 0.2, 2, 100, respectively. Four clusters were obtained and Cluster 4 was abandoned because it had only 3 nodes and contained no therapeutic targets. Cluster 3 was merged into cluster 2. Then cluster 1 and 2 were used to create the compounds−predicted targets−therapeutic targets interaction networks, respectively.

### Molecular docking

Crystal structures of protein targets were collected from the Protein Data Bank (PDB, http://www.rcsb.org/) [28] and inhibitors or agonists contained in the crystal structures were set as reference ligands. Besides, approved drugs or clinical trial drugs of the targets were collected from the Therapeutic Target Database and were also set as reference ligands (Additional file 2). Molecular docking experiments were performed at Molecular Operating Environment (MOE, 2018.01, Chemical Computing Group ULC). Pocket atoms were set as docking sites. A triangle matcher method was used as placement method. London dG scoring was used to select the top 30 poses (conformations and orientations of the ligand). Selected poses were refined in induced fit mode with the sidechains set as ‘free to move’ to achieve flexible receptor docking. GBVI/WSA dG scoring was finally used to select 5 poses with the lowest binding free energy, so a lower score means a more stable conformation.

### GO enrichment analysis

GO enrichment analysis was performed using ClueGO (version 2.5.2) [29] and CluePedia (version 1.5.2) [30]. GO biological process and GO molecular function were employed as the annotation sources and result show only enriched terms with *p* value < 0.01. Network was created with kappa statistics and reflected the similarity of the terms’ gene memberships. 0.4 kappa score was applied as threshold to create functional groups which were represented by their most significant term (leading term).

## Results

### Isolation and identification of LF triterpenoids

**Fig. 1:**
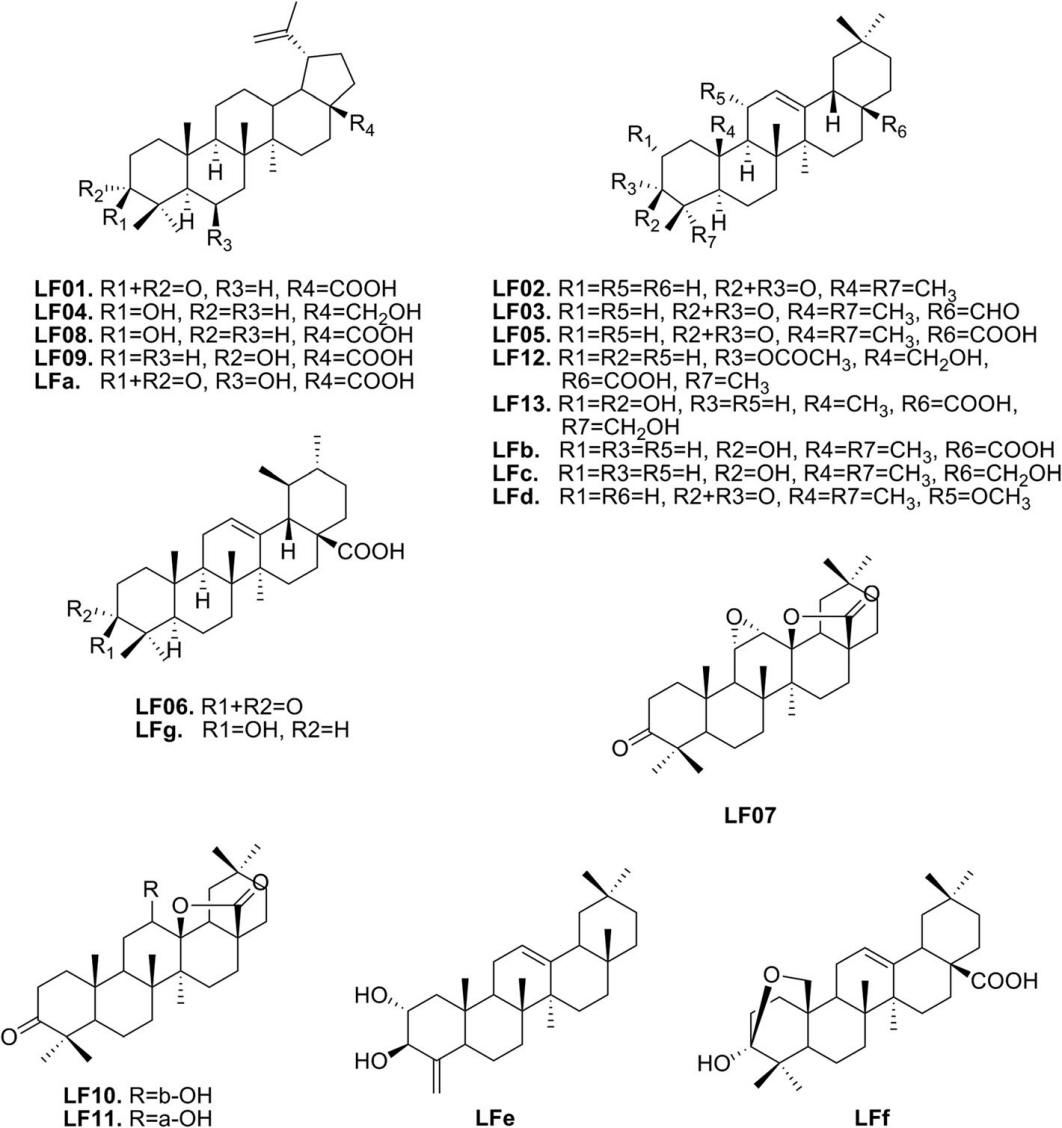
Chemical structures of the 20 triterpenoids contained in LF. **LF1** ~ **13** were isolated from LF and **LF03**, **LF04**, **LF05**, **LF08**, **LF09** and **LF10** were the first time isolated from LF. **LFa** ~ **g** were collected from literature

Thirteen triterpenoids were isolated from LF and were identified as betulonic acid (**LF01**), 28-nor-β-amyrenone (**LF02**), ambronal (**LF03**), betulin (**LF04**), oleanonic acid (**LF05**), ursonic acid (**LF06**), liquidambaric lactone (**LF07**), betulinic acid (**LF08**), epibetulinic acid (**LF09**), 3-oxo-12β-hydroxy-oleanan-28,13β-olide (**LF10**), hydroxyoleanonic lactone (**LF11**), 3α-acetoxy-25-hydroxyolean-12-en-28-oic acid (**LF12**), Arjunolic acid (**LF13**), respectively. Six of them, **LF03**, **LF04**, **LF05**, **LF08**, **LF09** and **LF10** (NMR spectrums were shown in Additional file 3), were the first time isolated from LF. Other known triterpenoids contained in LF, including 6β-Hydroxy-3-oxo-lup-20(29)-en-28-oic acid (**LFa**), Oleanolic acid (**LFb**), Erythrodiol (**LFc**), 11α-Methoxy-28-nor-β-amyrenone (**LFd**), 2α,3β-dihydroxy-23-norolean-4(24), 12(13)-dien-28-oic acid (**LFe**), lantanolic acid (**LFf**) and ursolic acid (**LFg**), were collected from literature [7–12]. Among the 20 triterpenoids, **LF01**, **LF04**, **LF08**, **LF09** and **LFa** belong to lupane-type triterpenoids, and **LF06**, **LFg** belong to ursane-type triterpenoids, and the others are oleanane-type triterpenoids (Fig. 1).

### Absorption and metabolism properties of LF triterpenoids

Predicted ADME properties indicated that LF triterpenoids were appropriate for oral administration as they were classified as HIA+ (human intestinal absorption was no less than 30%) and Caco2+ (Caco-2 permeability value (Papp) > 8×10^− 6^ cm/s) except **LF12** and **LF13** (HIA+, Caco2-). Although LF triterpenoids were labeled as P-glycoprotein substrates, **LF02**, **LF03**, **LF04**, **LF07**, **LF10**, **LF11** and **LFd** were also potential P-glycoprotein inhibitors, which might facilitate the transport of other triterpenoids. With respect to the predicted metabolism properties, all the 20 triterpenoids were the substrates of CYP450 3A4 and they did not inhibit the CYP450 metabolic activities.

### Exploration of anti-breast cancer targets of LF triterpenoids

**Fig. 2:**
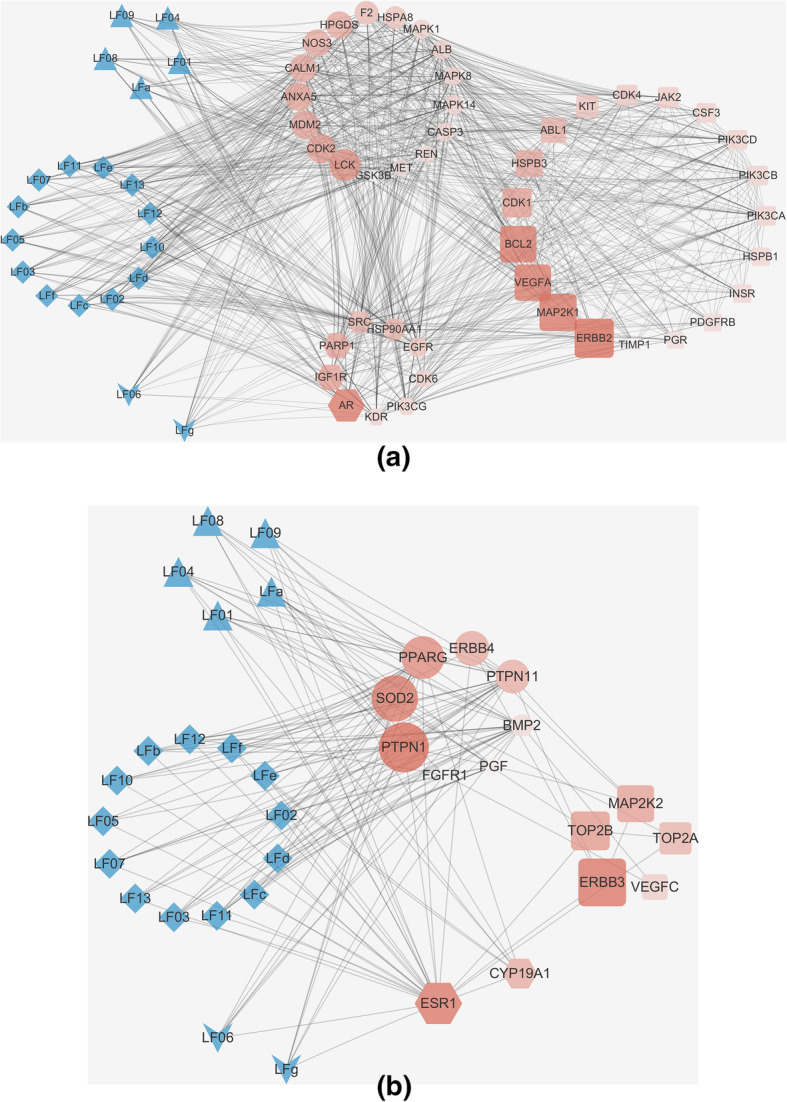
Compounds−predicted targets−therapeutic targets interaction networks 1 (**a**) and 2 (**b**). Protein targets are represented by their gene names; ▲ represents lupane-type triterpenoids; ♦ represents oleanane-type triterpenoids;  represents ursane-type triterpenoids; ● represents the predicted targets of LF triterpenoids; ■ represents the therapeutic targets of breast cancer;  represents the targets share by predicted targets and therapeutic targets. The layouts of each type of target nodes are determined by their MCODE score, and the setting is high values to big sizes and dark colors, indicating a more important role in the network

Potential targets of LF triterpenoids were ranked according to the normalized fit score, and the top 100 targets were selected for each compound, then these targets were merged and 174 targets were finally obtained. On the other hand, 111 therapeutic targets (including 27 subtypes) of breast cancer were collected (Additional file 4). Based on these targets, 2 optimized compounds−predicted targets−therapeutic targets interaction networks were constructed (Fig. 2). The network contained 36 predicted targets and 35 therapeutic targets, which had 11 targets in common. The result indicated that the 36 predicted targets remained in the network were more closely related to breast cancer progression than the others.

### Molecular docking

**Fig. 3:**
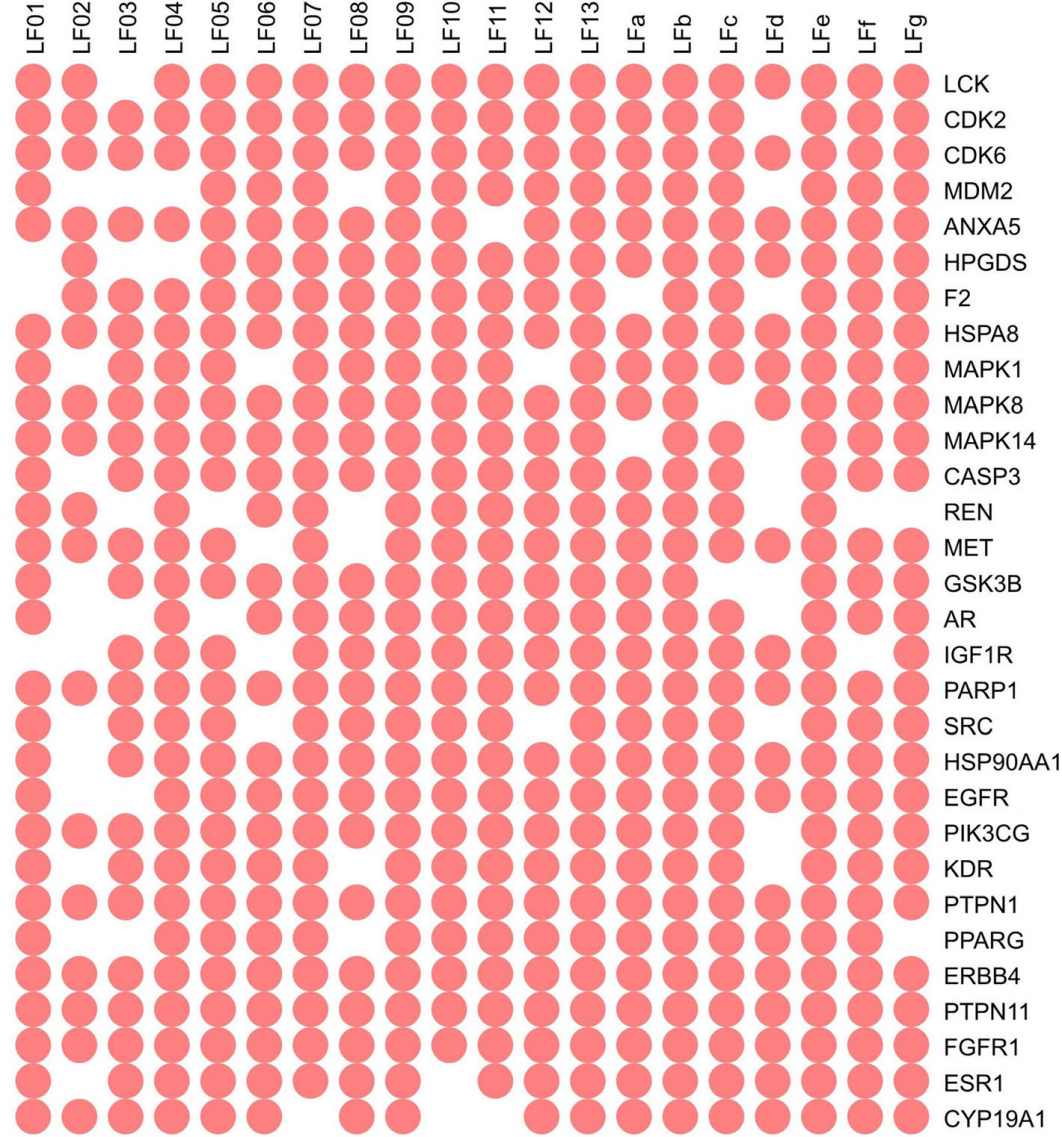
A brief summary of molecular docking result. Protein targets are represented by their gene names. Red spots means binding interactions exist between LF triterpenoids and targets

Molecular docking experiment was carried out on LF triterpenoids and the selected 36 targets except for calmodulin-1 (*CALM1*), eNOS (*NOS3*), serum albumin (*ALB*), Mn-SOD (*SOD2*), BMP2 and placenta growth factor (*PGF*) because no binding site for reference. Molecular docking results were summarized in Additional file 5 and Fig. 3. Eighteen of the 30 targets, including ErbB4 (*ERBB4*), EGFR, CDK2, CDK6, p38 MAPK (*MAPK14*), ERK2 (*MAPK1*), Lck (*LCK*), Src (*SRC*), IGF1R, PI3Kγ (*PIK3CG*), VEGFR2 (*KDR*), aromatase (*CYP19A1*), PTP1B (*PTPN1*), SHP2 (*PTPN11*), caspase-3 (*CASP3*), PARP-1, HSP90α (*HSP90AA1*) and HSC70 (*HSPA8*), were identified as the anti-breast cancer targets of LF triterpenoids. To acquire a better understanding about the results, binding characteristics and functions of these targets were analyzed as follows.

#### Targets that belong to protein kinases family

Half of the 30 targets belong to protein kinase superfamily, among which 14 targets were protein tyrosine kinases or CMGC serine/threonine protein kinases (Additional file 6). These two types of protein kinases play important roles in cancer progression and they are promising targets in breast cancer therapy [31, 32]. The catalytic domain of protein tyrosine kinases have high homology with that of serine/threonine protein kinase [33], which may explain why LF triterpenoids showed the features of ATP-competitive inhibitors targeting both the two types of protein kinases.

**Fig. 4:**
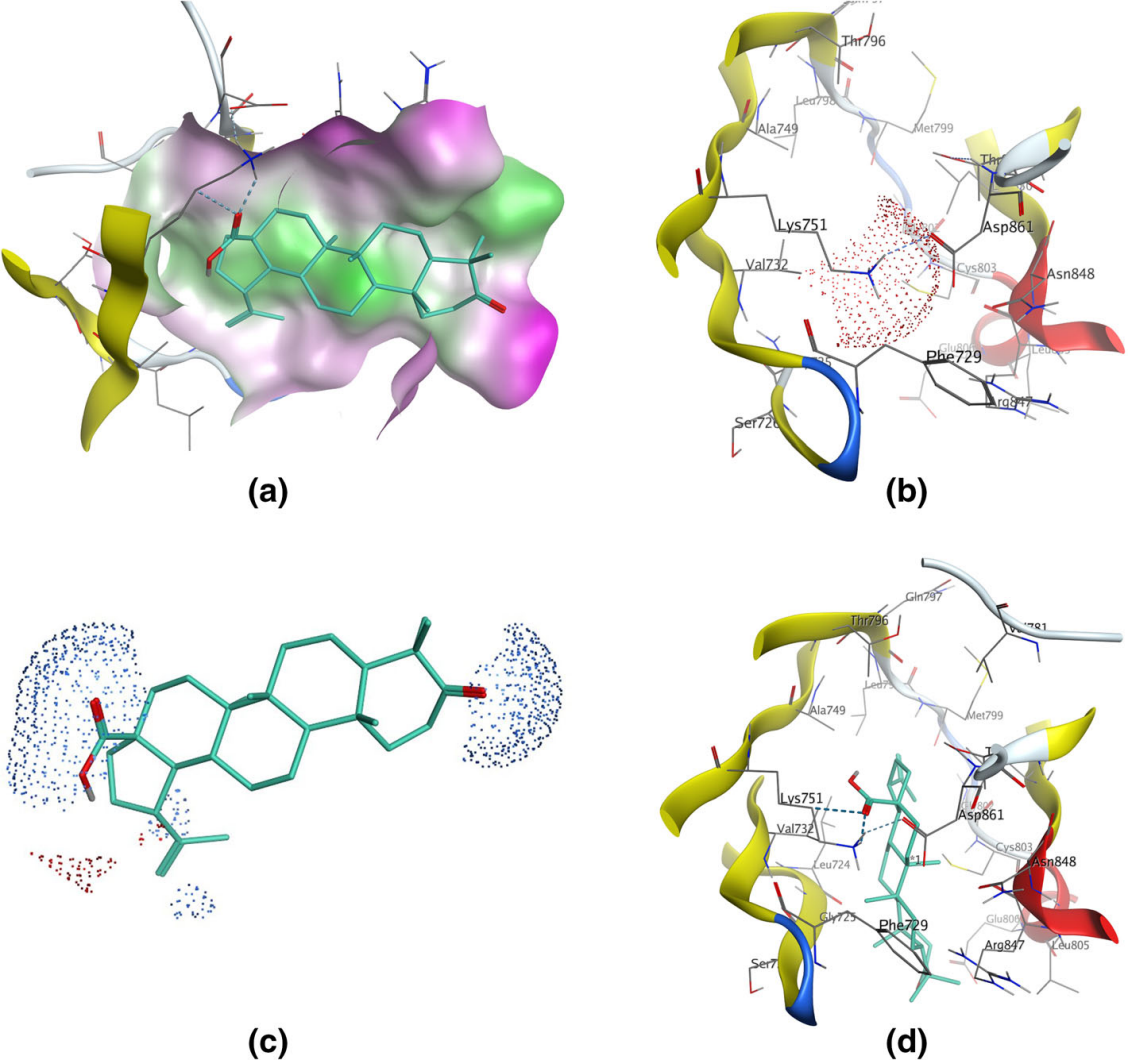
Binding interaction between **LF01** and ErbB4. **a** Molecular surface of the ATP binding pocket of ErBb4, colored by lipophilicity: green for hydrophobic, white for neutral and purple for hydrophilic regions. **b** Electrostatic map of Lys751 in the catalytic domain of ErbB4. Electrostatically preferred locations of hydrogen bond acceptor are colored red. **c** Electrostatic map of **LF01**. Electrostatically preferred locations of hydrogen bond donor are colored blue. **d** The 17-carboxyl group of **LF01** could form hydrogen bond with Lys751 in the binding site of ErbB4

EGFR subfamily members (belonging to receptor tyrosine kinases) play critical role in the pathogenesis of breast cancer. Besides the well-known EGFR (ErbB1/HER1) and ErbB2 (HER2, *ERBB2*), there is growing concern about another family member ErbB4 (HER4, *ERBB4*). Clinical studies suggested that to the patients with advanced stage or triple-negative breast cancer, increased expression of ErBb4 is associated with poor prognosis [34, 35]. Molecular docking result showed that hydrophobic skeleton is a favorable factor for LF triterpenoids to stabilize at the ATP binding site of ErbB4 (Fig. 4a). For active protein kinases, K/E/D/D signature motif plays important structural and catalytic roles [36]. It can be observed that Lys751 (the K of K/E/D/D in ErbB4) [37] formed a salt bridge with Asp861, therefore the -NH3^+^ group of Lys751 could be a stable hydrogen bond donor to LF triterpenoids (Fig. 4b). For most of the LF triterpenoids, hydrogen bond donor electrostatically preferred locations were at the opposite ends of skeleton: substituent located at C-17 and C-3 (Fig. 4c, Table 1). In the **LF01**-ErbB4 complex, it was the 17-carboxyl group bound to Lys751 (Fig. 4d); in the other LF triterpenoids-ErbB4 complexes, however, it was the C-3 substituent (Table 1). Among the three types of LF triterpenoids, lupane-type triterpenoids could form more stable hydrogen bonds with Lys751 because of the low binding energies, and the most stable hydrogen bond came from **LF01**. However, **LF04** could not form strong interaction with Lys751 might because the 17-carboxyl group was displaced by hydroxymethyl group. LF triterpenoids bound to EGFR in a similar fashion, but Lys745 (the K of K/E/D/D in EGFR) [37] bound to the 17-carboxyl group more often than the C-3 substituent, and the most stable hydrogen bonds came from **LF05** and **LFg** (Table 1). However, lupane-type triterpenoids did not show strong interactions with EGFR, which meant that lupane-type triterpenoids from LF might have higher affinity and inhibition to ErbB4 than EGFR.

**Table 1:** Binding interactions between LF triterpenoids and Lys751 of ErbB4/Lys 745 of EGFR

Skeleton	Compounds	Binding with Lys751 of ErbB4			Binding with Lys745 of EGFR		
		Substituent	E^**a**^ (kcal/mol)	Scores^**b**^	Substituent	E^**a**^ (kcal/mol)	Scores^**b**^
Lupane-type	LF01	17-COOH	−9.8	− 5.9640	17-COOH	− 2.1	−7.0066
	LF04	3β-OH	− 0.8	−5.6831	3β-OH	−0.6	− 6.2663
	LF08	3β-OH	−8.3	−6.1819			
	LF09	3α-OH	−5.4	−6.3759			
	LFa	3 = O	−6.3	−5.4535			
Oleanane-type	LF03	3 = O	− 2.4	−5.8667			
	LF05	3 = O	−4.3	−5.8143	17-COOH	−11.2	−6.6884
	LF07	3 = O	−2.3	−5.9734			
	LF11				12α-OH	−1.4	−6.3773
	LF12	3α-OCOCH_3_	−6.1	5.9681	17-COOH	−2.1	−6.9071
	LF13	3β-OH	−5.3	−6.2568	17-COOH	−0.8	−6.7261
	LFb				17-COOH	−1.1	−6.8580
	LFc	3β-OH	−8.0	−5.9229			
	LFd	11-OCH_3_	−4.9	−6.1058			
	LFe	3β-OH	−0.6	−5.5257			
	LFf	3α-OH	−1.4	−5.7394	3α-OH	−4.5	−5.9924
Ursane-type	LFg				17-COOH	−9.0	−6.9074

^a^ The lowest binding energy; ^b^ Scores of the conformations with the lowest binding energy

CDKs (belonging to CMGC serine/threonine protein kinase family) are also important anti-breast cancer targets. Although selective CDK4/6 inhibition results in less toxicity, a huge challenge is that breast cancer (including basal-like breast cancer) adapt quickly to CDK4/6 inhibition, while inhibiting CDK2 simultaneously is an effective solution [38, 39]. Molecular docking result showed that most LF triterpenoids could bind with both CDK6 and CDK2. In LF triterpenoids-CDK6 complexes, binding sites were catalytic loop (especially was Lys147) and activation segment (especially was Asp163, the D of DFG and the second D of K/E/D/D). In LF triterpenoids-CDK2 complexes, however, binding sites mainly were hinge residues (Asp86, Gln85, His84 and Leu83) and the K of K/E/D/D (Lys33) (Table 2). Because hinge region played an important role in inhibitor binding [40], the result indicated that LF triterpenoids probably had a higher inhibitory activity to CKD2 than CDK6.

**Table 2:** Binding site of LF triterpenoids in CDK2 and CDK6

Skeleton	Compounds	Binding site in CDK2	Binding site in CDK6
Lupane-type	LF01	hinge residues, catalytic loop	catalytic loop
	LF04	hinge residues	catalytic loop, activation segment
	LF08		catalytic loop, activation segment
	LF09	K of K/E/D/D	catalytic loop
	LFa	hinge residues, K of K/E/D/D	catalytic loop
Oleanane-type	LF02	K of K/E/D/D	catalytic loop
	LF03	hinge residues, K of K/E/D/D	catalytic loop
	LF05	hinge residues, catalytic loop, activation segment	catalytic loop
	LF07	hinge residues	catalytic loop
	LF10	hinge residues	catalytic loop, activation segment
	LF11	hinge residues	catalytic loop, activation segment
	LF12	hinge residues, K of K/E/D/D	catalytic loop
	LF13	hinge residues, K of K/E/D/D	catalytic loop
	LFb	hinge residues, K of K/E/D/D, catalytic loop	catalytic loop
	LFc	hinge residues, K of K/E/D/D, catalytic loop	
	LFd		catalytic loop
	LFe	catalytic loop, activation segment	activation segment
	LFf	K of K/E/D/D, catalytic loop	activation segment
Ursane-type	LF06		activation segment
	LFg	hinge residues, K of K/E/D/D	catalytic loop

It was reported that phosphorylated MAPKs including ERK2 (*MAPK1*), JNK1 (*MAPK8*) and p38 MAPK (*MAPK14*) over-expressed in breast cancer patients and is associated with poor prognosis [41–43]. Molecular docking result showed that LF triterpenoids could bind to the hinge region (Thr106, His107, Met109 and Gly110) and the DFG motif (Asp168 and Phe169) of p38 MAPK, but in LF triterpenoids-ERK2 complexes the interactions were weaker, and LF triterpenoids could not form hydrogen bonds with the hinge region of JNK1 (Table 3), indicating LF triterpenoids had higher inhibitions to p38 MAPK than ERK2 and JNK1. Lck (*LCK*) plays a pathological role in breast cancer progression probably by activating MAPK signaling pathway [44–46]. Molecular docking result showed that LF triterpenoids could bind with key residues of Lck including the conserved ATP binding lysine (Lys273), the linker sequence between the SH2 domain and the catalytic domain (Ser323), the catalytic domain (Ala368 and Asn369) and the activation loop (Asp382, Glu390, Tyr394, Thr395, and Arg397), which meant that LF triterpenoids could compete with ATP for binding Lck and inhibit its activity.

**Table 3:** Binding interactions between LF triterpenoids and the hinge region of p38 MAPK/ERK2

Skeleton	Compounds	Binding with p38 MAPK		Binding with ERK2	
		E^**a**^ (kcal/mol)	Scores^**b**^	E^**a**^ (kcal/mol)	Scores^**b**^
Lupane-type	LF01	− 2.9 ~ − 3.2	− 7.6148 ~ − 7.6724	− 0.7 ~ − 1.5	− 6.7461 ~ − 6.9596
	LF04	− 1.1 ~ − 1.7	−7.3417 ~ − 7.9353	− 1.0 ~ − 1.3	− 7.0514 ~ − 7.0586
	LF08	−0.7 ~ − 1.6	− 7.7455 ~ − 8.1666	− 1.0	−7.0391
	LF09	−1.5	− 8.3157	−1.1 ~ − 2.8	− 6.7530 ~ − 6.8403
Oleanane-type	LF05	−1.8 ~ − 2.9	− 7.7640 ~ − 8.0353		
	LF07			− 0.9	−6.5656
	LF10			−1.2 ~ − 1.7	−6.9311 ~ −7.5512
	LF11	−1.0	−7.1710		
	LF12	−0.7	−8.3375		
	LF13	−1.4 ~ − 2.5	− 8.2020 ~ − 8.2068		
	LFb	−1.3 ~ − 2.6	−7.3082 ~ − 7.7737		
	LFe	−0.7	−7.8543	−1.0 ~ − 1.9	−6.4379 ~ −6.9347
	LFf	− 1.3 ~ − 4.3	− 7.9048 ~ −8.1671		
Ursane-type	LF06	−2.3	−7.9031		
	LFg	−1.4	−8.0787		

^a^ binding energy; ^b^ Scores of the conformations that binding with the hinge region of p38 MAPK or ERK2

In addition, Src (*SRC*), VEGFR2 (*KDR*), IGF1R and PI3Kγ (*PIK3CG*) have already been successful targets in breast cancer therapy. Molecular docking result showed that LF triterpenoids could bind with these protein kinases and act as ATP competitive inhibitors.

#### Targets that belong to nuclear hormone receptor family and cytochrome P450 family

For nuclear hormone receptors ERα (*ESR1*), AR and PPARγ (*PPARG*), both agonist and antagonist bind at the same site of LBD, therefore it’s hard to figure out whether the ligand were agonist or antagonist through molecular docking result. Besides, AR and PPARγ are still complicated targets for breast cancer treatment because studies gave contradictory conclusions [47, 48]. Hence effects of LF triterpenoids caused by binding with these 3 targets need further studies. Human cytochrome P450 aromatase (AROM, *CYP19A1*) use androgens as substrates with high specificity and catalyze them to estrogens [49], and its inhibitors, such as exemestane, were used to treat breast cancer for many years. Molecular docking result showed that LF triterpenoids could compete with testosterone for binding aromatase, forming hydrogen bonds with Met374 and Arg115 in the catalytic cleft, and it is remarkable that LF triterpenoids had more stable conformations (with scores from − 8.1861 to − 9.3923) than testosterone (with scores from − 6.5824 to − 7.8243) and exemestane (scores − 7.7675) in the binding pocket, indicating that LF triterpenoids might be promising inhibitors of aromatase.

#### PTPs

**Fig. 5:**
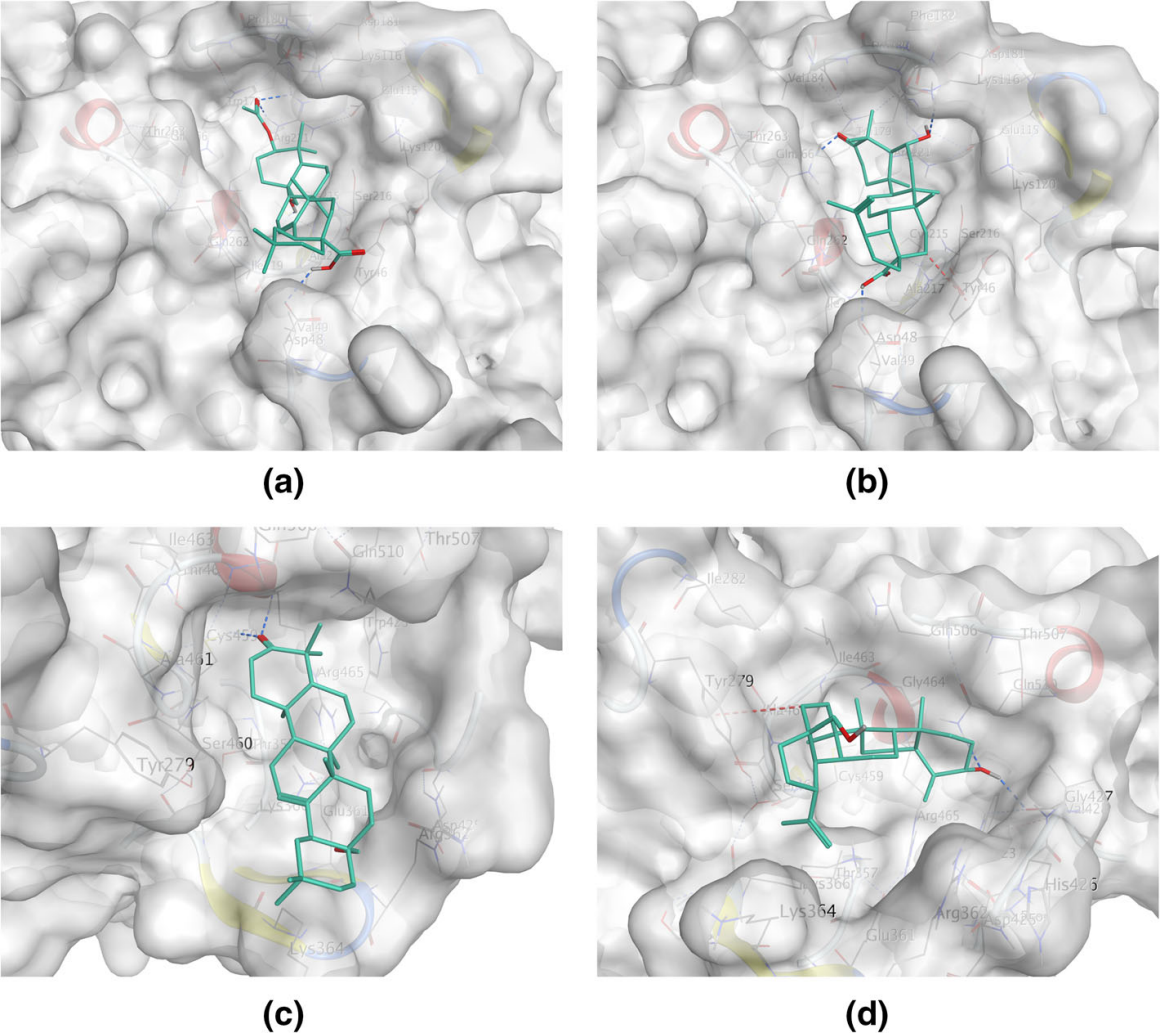
Binding sites of LF triterpenoids on PTP1B and SHP2. **a LF12** could bind with PTP1B at Arg221 on A site, and Asp48 on C site; **b LFa** could bind with PTP1B at Gln266 on A site, Tyr46 and Asp48 on C site, and Lys116 on D site; **c LF03** could bind with SHP2 at Cys459 and Arg465 in P-loop; **d LF04** could bind with SHP2 at Gly427 in WPD loop, and Tyr279 in *p*Tyr recognition loop

Although PTP1B (*PTPN1*) was previously known could dephosphorylate some receptor protein tyrosine kinases, studies showed that inhibition of PTP1B could be a new therapeutic strategy for breast cancer, including triple-negative breast cancer [50, 51]. The catalytic domain of PTP1B can be divided into four sites and A site is catalytic pocket. However because of the high homology of the catalytic domain between PTP1B and TCPTP (a phosphatase related to regulating T-cell activation), inhibitors only binding to A site have low selectivity [52, 53]. Molecular docking result showed that LF triterpenoids could bind on A site (Ala217, Gly220, Arg221, Gln266), C site (Tyr46, Arg47 and Asp48) and D site (Glu115, Lys116, Asp181). Lys116 on D site was a preferred residue to half of LF triterpenoids, such as **LF05**, **LF06** and **LF12** (the lowest binding energy was-9.1, − 8.3 and − 9.0 kcal/mol, respectively), but could not form bond with LF triterpenoids without 17-carboxyl group. Nearly half of LF triterpenoids could bind with more than one site, such as **LF12** (occupied AC sites) and **LFa** (occupied ACD sites) (Fig. 5a, b). These characters of LF triterpenoids probably could increase their selectivity and inhibitory activity to PTP1B.

SHP2 (*PTPN11*) is required for the full activation of MAPK signaling pathway and could promotes breast cancer (including triple-negative breast cancer) progression [54, 55]. Important binding sites for SHP2 inhibitors include P-loop (the PTP signature motif for recognition of phosphoryl moiety in substrate), WPD loop (lid of the active site pocket) and *p*Tyr recognition loop [56]. Molecular docking result of SHP2 showed that LF triterpenoids could bind with Cys459 and Arg465 in P-loop (such as **LF03**), and Gly427 in the flexible WPD loop, and form C-H···π interaction with Tyr279 in *p*Tyr recognition loop (such as **LF04**) (Fig. 5c, d). Besides, about half of LF triterpenoids could form hydrogen bond with the side chain of Lys366, such as **LF05**, **LF12** and **LFg** (the lowest binding energy was − 8.5, − 8.2 and − 9.9 kcal/mol, respectively).

#### Targets related to apoptosis

**Fig. 6:**
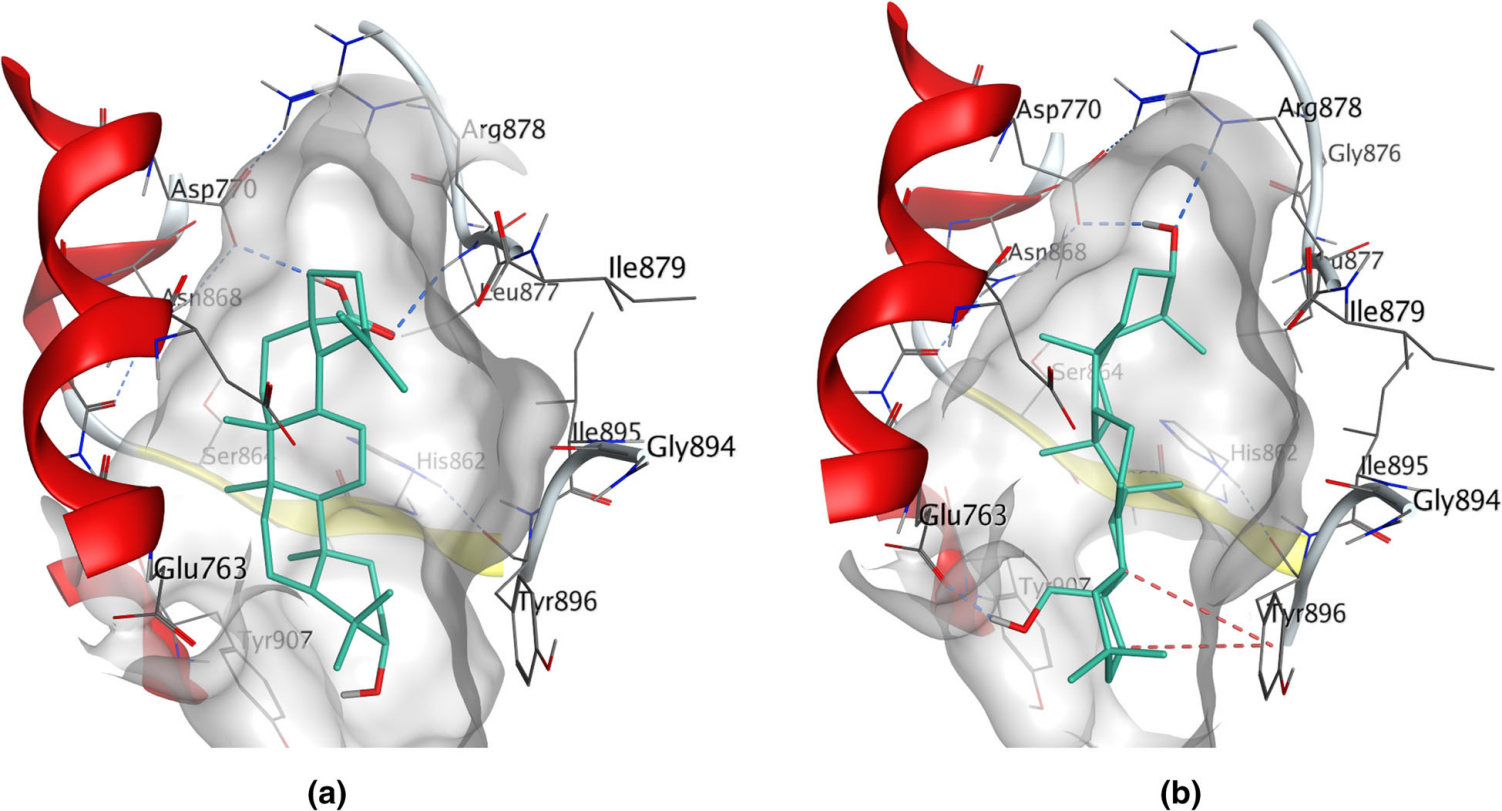
Binding sites of LF triterpenoids on PARP1. **a LF08** could form hydrogen bound with the backbone of Arg878 and the sidechain of Asp770 on AD site; **b** Besides binding on AD site, **LFc** could form C-H···π interaction with Tyr896 on NI site, and form hydrogen bound with Glu763 on PH site

Although the proteolytic enzyme caspase-3 (*CASP3*) could initiates apoptosis by cleaving and inactivating PARP1, it also could stimulate survived breast cancer cell growth by promoting PGE2 release [57]. Molecular docking result showed that LF triterpenoids could bind with caspase-3 like its inhibitors. However, LF triterpenoids could promote apoptosis by directly inhibiting PARP1. PARP1 is a base excision repair protein that could promote cancer cell survival, and is an effective target in triple-negative breast cancer [58, 59]. Molecular docking result showed that C-17 substituent of LF triterpenoids could form hydrogen bound with the backbone of Arg878 (or the sidechain of Asp770) on adenine ribose binding site (AD site) of PARP1, such as **LF08** (Fig. 6a). And binding the AD site is considered to gain selectivity for the inhibitors [60]. Besides, some LF triterpenoids could form C-H···π interaction with Tyr896 in nicotinamide binding pocket (NI site), or form hydrogen bound with Asp766 and Glu763 on phosphate binding site (PH site), such as **LFc** (Fig. 6b). The result indicated that LF triterpenoids could compete with NAD^+^ for binding PARP1 then inhibit its activity.

Molecular chaperones HSP90α (*HSP90AA1*) and HSC70 (*HSPA8*) could assist the stability and function of many oncogenic proteins then keep cancer cells surviving [61]. Molecular docking result showed that LF triterpenoids could bind with HSP90α and HSC70 at ATP binding site, indicating they may act as HSPs’ inhibitors then promote cell death.

The p53 protein can initiate apoptosis while its negative regulator MDM2 could bind and inactive p53 thus acts as a tumor promoter [62]. Molecular docking result showed that LF triterpenoids could occupy the hydrophobic pockets of MDM2 that bind with p53, and formed hydrogen bonds with residues Met62, Tyr67, Tyr100, or formed C-H···π/O-H···π interactions with His96, but these interactions were not strong enough (binding energy − 0.6 ~ − 2.9 kcal/mol) to compete with the macromolecule p53, indicating LF triterpenoids might be not high affinity MDM2 antagonists.

#### Other targets

HPGDS catalyzes the conversion of PGH_2_ to PGD_2_, and closely related to the progression of inflammation. Although its role in breast cancer is not entirely clear, it is reported that HPGDS suppress gastrointestinal cancer progression [63, 64]. However, molecular docking result showed that LF triterpenoids (except for **LF01** and **LF04**) could form hydrogen bonds and C-H···π/O-H···π interactions with Arg14 and Trp104 of HPGDS, which are important residues for binding affinity and binding pocket stability, indicating LF triterpenoids could compete with PGH_2_ for binding HPGDS and acting as an inhibitor.

For other targets including annexin A5 (*ANXA5*), prothrombin (*F2*) and renin (*REN*), there was no firm evidence to link their functions directly to breast cancer progression, and they were less significant in the PPI network, which meant that they might be not important targets for LF triterpenoids.

### GO enrichment analysis

**Fig. 7:**
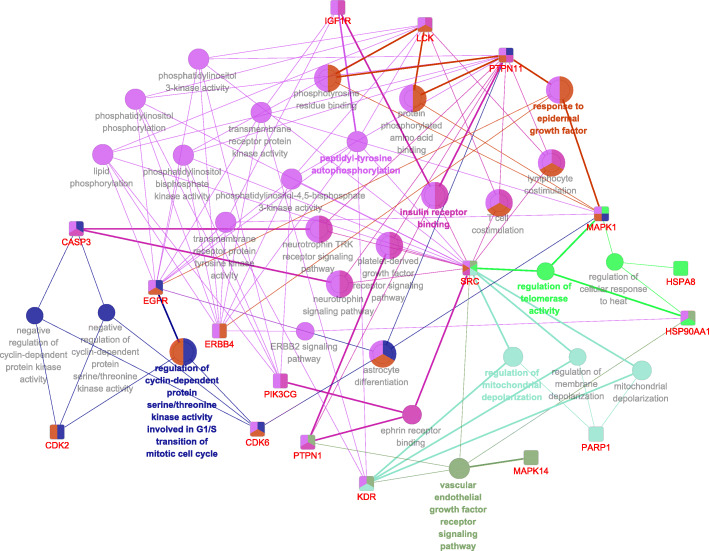
GO enrichment analysis of the identified targets of LF triterpenoids. Protein targets are represented by their gene names. Functional groups are colored and represented by their most significant term (leading term) based on the kappa statistics. The edge thickness shows kappa-score relations between the targets and terms

GO enrichment analysis was conducted on the 18 identified targets according to the molecular docking result. As a result, totaling 29 terms were acquired, and they could be clustered into 7 functional groups (Additional file 7). The biggest group that covered 44.19% of enriched terms was peptidyl-tyrosine autophosphorylation group, indicating that inhibition of protein tyrosine kinases autophosphorylation might be the primary mechanism for the anti-breast cancer effect of LF triterpenoids. Figure 7 revealed that the most relevant targets for peptidyl-tyrosine autophosphorylation functional group were ErbB4 and EGFR, because they had more connections (each had 9 edges) with terms belonging to this group than the other targets. Besides, targets of LF triterpenoids also involved in response to epidermal growth factor, regulation of cyclin-dependent protein serine/threonine kinase activity, insulin receptor binding, regulation of mitochondrial depolarization, regulation of telomerase activity, vascular endothelial growth factor receptor signaling pathway, which were closed related to breast cancer progression.

**Fig. 8:**
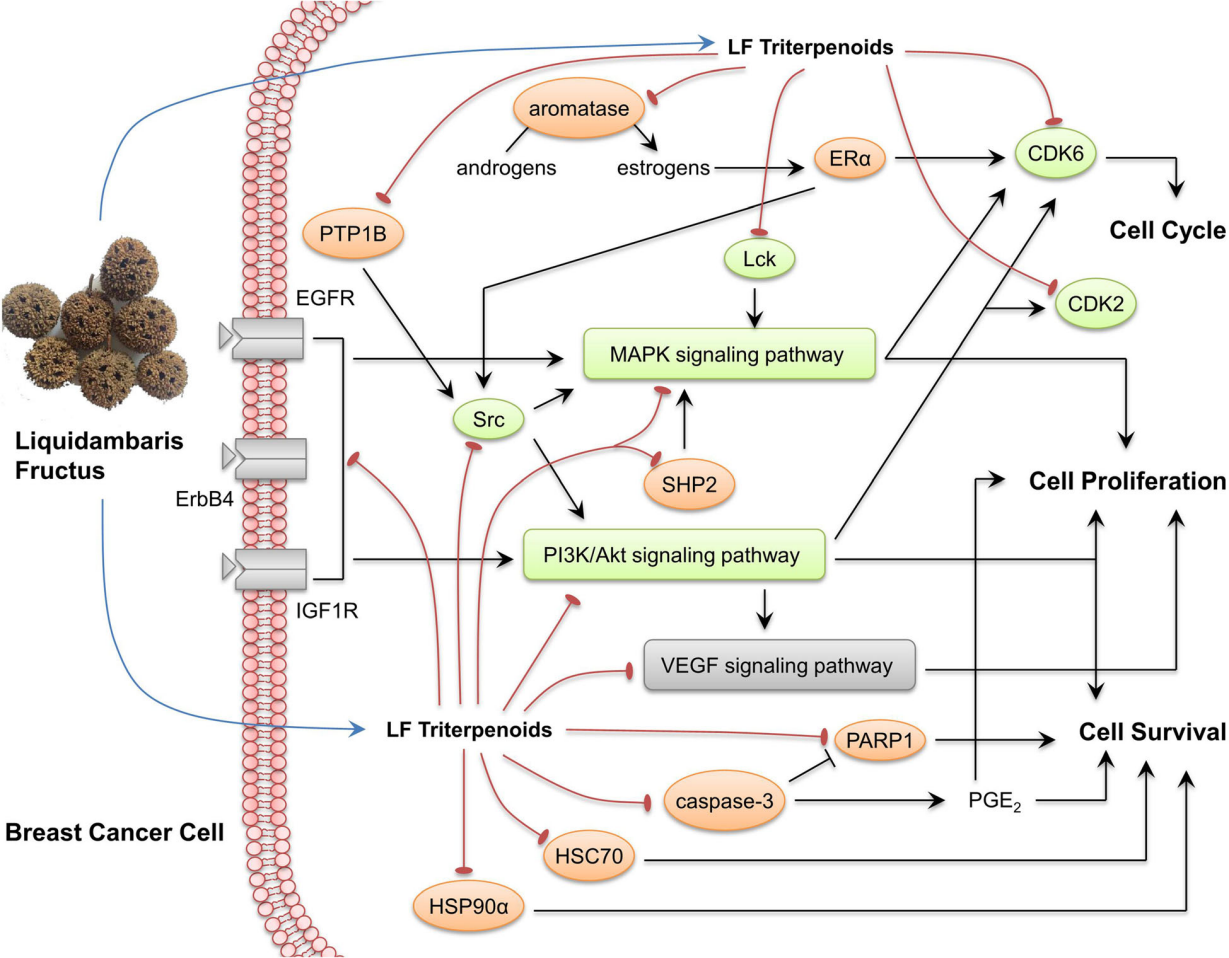
Predicted mechanism of the anti-breast cancer effect of LF triterpenoids

According to the above result, we then analyzed signaling pathways concerned with the identified targets based on related studies [45, 46, 50, 51, 55, 57] and KEGG pathway database. By binding with the identified targets, LF triterpenoids could inhibit ErbB signaling pathway, MAPK signaling pathway, PI3K/Akt signaling pathway and VEGF signaling pathway, then inhibit cancer cells proliferation, and induce the cell cycle arrest and apoptosis (Fig. 8), which could explain the result of our previous research [6]. There have been studies showing that betulonic acid and other LF triterpenoids could induce cancer cell apoptosis and cell cycle arrest via inhibiting PI3K/Akt pathway [65–67], which could also support the predicted mechanism.

## Discussion

According to the concept of polypharmacology, for complex diseases like cancers, inhibition of single target often induces chemo-resistance and lead to therapy failure [68–70]. In previous study we found the anti-breast cancer effect of betulonic acid [6], and this study revealed that LF triterpenoids might exert anti-breast cancer effect by directly inhibit multiple protein targets and signaling pathways, especially ErbB4 and EGFR and related pathways. It is worth noting that many of these targets were investigated or potential therapeutic targets of triple-negative, basal-like or advanced stage breast cancers, and patients with these types of breast cancer have rapid progression, high mortality rates, and high risk of recurrence and metastasis [59, 71]. Therefore the multi-targeted activity of LF triterpenoids revealed in this study could bring some inspirations in breast cancer treatment (Additional file 8).

In addition, this study left an extra hint about the traditional applications of LF: as a high binding affinity target of LF triterpenoids, ErbB4 activity is essential for mammary gland development and the initiation of lactation [72], which meant that LF might aggravate rather than alleviate hypogalactia, contradicted with the description in Chinese Pharmacopoeia. Actually in early literature that involved LF, such as *Bencao Gangmu Shiyi* (1765 AD), no record showed that LF could promote lactation. Therefore this usage probably was an extension of its original efficacy because hypogalactia is considered a symptom caused by blocking of meridians. However, this extended usage need more evidence and this study gave a negative conclusion (Additional file 8).

Compared to synthetic drugs, Traditional Chinese Medicine is generally considered to have better safety. Although LF has been used for more than 250 years and no obvious adverse effects were reported, based on the result of the present study, there are still some precautions should be noted. Because ErbB4 play essential roles in the development of cardiac muscle and central nervous system during pregnancy [73], LF triterpenoids might have adverse effect on fetal development by inhibiting the activity of ErbB4. Besides, because Lck activation could initiate T cell antigen receptor signaling, and is a critical step in immune response [74], therefore LF triterpenoids probably could cause immune suppression by inhibiting Lck, and should be used very carefully on patients with immunodeficiency.

## Conclusion

To further investigate the chemical ingredients of LF, 13 triterpenoids were identified and 6 of them were the first time isolated from LF. Using network pharmacology-based analysis in combination with molecular docking method, 18 protein targets, ErbB4, EGFR, CDK2, CDK6, p38 MAPK, ERK2, Lck, Src, IGF1R, PI3Kγ, VEGFR2, aromatase, PTP1B, SHP2, caspase-3, PARP-1, HSP90α and HSC70, were identified and by binding with these targets, LF triterpenoids could inhibit ErbB signaling pathway, MAPK signaling pathway, PI3K/Akt signaling pathway and VEGF signaling pathway, then inhibit breast cancer cells proliferation, and induce the cell cycle arrest and apoptosis. GO enrichment analysis indicated that ErbB4 and EGFR and related pathways might play more important roles during the process.

On the other hand, this study also revealed that LF triterpenoids could inhibit the activity of ErbB4 and accordingly suppressed rather than promoted lactation. Therefore the traditional applications of LF on hypogalactia should be reassessed systematically. Besides, this study also indicated that LF must be used very cautiously to treat women during pregnancy or patients with immunodeficiency, because LF triterpenoids might cause embryo toxicity and immune suppression by inhibiting the activity of ErbB4 and Lck.

## Supplementary Information

**Additional file 1.** Separation process of the chemical compounds in LF.

**Additional file 2.** Protein crystal structures and reference ligands used in the molecular docking experiment.

**Additional file 3.** The NMR spectrum of LF03, LF04, LF05, LF08, LF09 and LF10.

**Additional file 4.** Predicted targets of LF triterpenoids and therapeutic targets of breast cancer.

**Additional file 5.** Molecular docking results of LF triterpenoids.

**Additional file 6.** Protein families that the molecular docking targets belonged to.

**Additional file 7.** Functionally grouped network view of the GO enrichment analysis result.

**Additional file 8.** The framework and research strategy of the article.

## Data Availability

The datasets used and analyzed during the current study are available from the corresponding author on reasonable request.

## Abbreviations

AR: Androgen receptor
CDKs: Cyclin-dependent kinases
CMGC: The combination of CDKs
MAPKs: GSKs and CLKs
DFG: Asp-Phe-Gly
EGFR: Epidermal growth factor receptor
ERα: Estrogen receptor α
ERK2: Extracellular signal-regulated kinase 2
HPGDS: Hematopoietic prostaglandin D synthase
K/E/D/D: Lys/Glu/Asp/Asp
LBD: Ligand-binding domain
Lck: Lymphocyte cell-specific protein-tyrosine kinase
MAPK: Mitogen-activated protein kinase
PTP: Protein tyrosine phosphatases
SHP2: Src-homology 2 domain-containing phosphatase 2
TCPTP: T-cell protein tyrosine phosphatase
