# Supplementary material for: Identification of the anti-breast cancer targets of triterpenoids in Liquidambaris Fructus and the hints for its traditional applications
Source: BMC Complement Med Ther. 2020 Nov 27;20:369. doi: 10.1186/s12906-020-03143-8 (PMC7694930; doi:10.1186/s12906-020-03143-8)
Supplement: Supplementary file 1 — Additional file 1. Separation process of the chemical compounds in LF. [file 12906_2020_3143_MOESM1_ESM.docx]

Recrystallized in CHCl_3_− MeOH (2:1)

50 : 4

Recrystallized in EtOAC − acetone (2:1)

Recrystallized in CHCl_3_− MeOH (2:1)

PTLC, CH2Cl2 −MeOH− AcOH (15:1:0.3)

Sephadex LH-20 CC, eluted with CHCl_3_− MeOH (1:1)

Recrystallized in CHCl_3_− MeOH (2:1)

Sephadex LH-20 CC, eluted with CHCl_3_− MeOH (1:1)

EtOAc extraction of LF (120g)

Silica gel CC, eluted with cyclohexane−EtOAc

50 : 1

Fr. A

Fr. B (6.2g)

Fr. C (15.6g)

50 : 2

**LF02**

(58mg)

Silica gel CC, eluted with cyclohexane –EtOAc 30:1→30:5

Fr. C1

Fr. C2 (0.9g)

Fr. C3~5

Silica gel CC, eluted with cyclohexane –EtOAc 20:1

**LF03**

(64mg)

**LF04**

(26mg)

Fr. D (25.3g)

Silica gel CC, eluted with cyclohexane –EtOAc 25:1→25:5

Fr. D1~2

Fr. D3 (8.2g)

Fr. D4

Silica gel CC, eluted with cyclohexane –EtOAc 25:1→25:5

**LF01**

(130mg)

**LF05**

(74mg)

**LF06**

(40mg)

Fr. E (17.4g)

Silica gel CC, eluted with CH_2_Cl_2_ – EtOAc 30:1→20:1

Fr. E1

Fr. E2

(2.4g)

Sephadex LH-20 CC, eluted with CHCl_3_− MeOH (1:1)

**LF07**

(48mg)

**LF08**

(21mg)

**LF09**

(34mg)

Fr. E3

Fr. E4

(1.8g)

Fr. E5

Sephadex LH-20 CC, eluted with CHCl_3_− MeOH (1:1)

**LF10**

(41mg)

**LF11**

(24mg)

50 : 5

Fr. F (7.9g)

Fr. F1

Fr. F2 (2.3g)

Fr. F3

Silica gel CC, eluted with CH_2_Cl_2_ – EtOAc 20:1

**LF12**

(23mg)

50 : 8

Fr. F (20.2g)

Silica gel CC, eluted with CH_2_Cl_2_ – EtOAc 15:1

Fr. G1

Fr. G2 (1.5g)

Fr. G3

Mixture

**LF13**

(35mg)

.

**Additional Fig. 1** Separation process of the chemical compounds in LF
