# Supplementary material for: Identification of the anti-breast cancer targets of triterpenoids in Liquidambaris Fructus and the hints for its traditional applications
Source: BMC Complement Med Ther. 2020 Nov 27;20:369. doi: 10.1186/s12906-020-03143-8 (PMC7694930; doi:10.1186/s12906-020-03143-8)
Supplement: Supplementary file 2 — Additional file 2. Protein crystal structures and reference ligands used in the molecular docking experiment. [file 12906_2020_3143_MOESM2_ESM.docx]

**Additional Table 1** Protein crystal structures and reference ligands used in the molecular docking experiment

| **Protein target** | **Crystal structure*** | **Binding site** | **Ligand** | **Ligand type** | **Disease** |
| --- | --- | --- | --- | --- | --- |
| Lck  (*LCK*) | 3MPM | ATP binding site | Dasatinib | Approved drug | Chronic myelogenous leukaemia |
|  |  |  | 5LK* | Inhibitor |  |
| CDK2 | 2R3I | ATP binding site | *R*-roscovitine | Clinical trial drug | Non-small-cell lung cancer |
|  |  |  | SCF* | Inhibitor |  |
| CDK6 | 5L2T | ATP binding site | Ribociclib (6ZZ*) | Approved drug | Breast cancer |
| MDM2 | 4QOC | Inhibitor binding site | AMG 232 | Clinical trial drug | Solid tumour/cancer |
|  |  |  | 35T* | Inhibitor |  |
| Annexin A5  (*ANXA5*) | 1HAK | Inhibitor binding site | K21* | Inhibitor |  |
| HPGDS | 2CVD | PGH2 binding site | HQL* | Inhibitor |  |
| Prothrombin  (*F2*) | 1G32 | Inhibitor binding site | Dabigatran | Approved drug | Stroke |
|  |  |  | R11* | Inhibitor |  |
| HSC70  (*HSPA8*) | 3FZH | ATP binding site | 3BH* | Inhibitor |  |
| ERK2  (*MAPK1*) | 3SA0 | ATP binding site | Norathyriol (NRA*) | Natural compound (Inhibitor) |  |
| JNK1  (*MAPK8*) | 3ELJ | ATP binding site | GS7* | Inhibitor |  |
| p38 MAPK  (*MAPK14*) | 4EH3 | ATP binding site | Naringenin (NAR*) | Natural compound (Inhibitor) |  |
| caspase-3  (*CASP3*) | 3KJF | Inhibitor binding site | B92* | Inhibitor |  |
| Rennin  (*REN*) | 2V0Z | Inhibitor binding site | Aliskiren (C41*) | Approved drug | Hypertension |
| c-Met  (*MET*) | 2WGJ | ATP binding site | Crizotinib (VGH*) | Approved drug | Non-small-cell lung cancer |
| GSK-3β  (GSK3B) | 1Q5K | ATP binding site | TMU* | Inhibitor |  |
| AR | 2AMA | Ligand binding domain | Dromostanolone | Approved drug | Mammary tumour |
|  |  |  | Dihydrotestosterone (DHT*) | Agonist |  |
| IGF1R | 3I81 | ATP binding site | OSI-906 | Clinical trial drug | Solid tumour/cancer |
|  |  |  | EBI* | Inhibitor |  |
| PARP1 | 4L6S | NAD+ binding site | KU-0058948 | Approved drug | Ovarian cancer |
|  |  |  | 1WQ* | Inhibitor |  |
| Src  (*SRC*) | 4MXO | ATP binding site | Bosutinib (DB8*) | Approved drug | Breast cancer |
| HSP90α  (*HSP90AA1*) | 3WHA | ATP binding site | Tanespimycin | Clinical trial drug | Breast cancer |
|  |  |  | WHA* | Inhibitor |  |
| EGFR | 1XKK | ATP binding site | Lapatinib (FMM*) | Approved drug | Breast cancer |
| PI3Kγ  (*PIK3CG*) | 3SD5 | ATP binding site | Buparlisib (SD5*) | Clinical trial drug | Breast cancer |
| VEGFR2  (*KDR*) | 3WZD | ATP binding site | Apatinib | Approved drug | Breast cancer |
|  |  |  | Lenvatinib (LEV*) | Approved drug | Thyroid cancer |
| PTP1B  (*PTPN1*) | 3EAX | Inhibitor binding site | LZP* | Inhibitor |  |
| PPARγ  (*PPARG*) | 5Y2O | Ligand binding domain | Pioglitazone (8N6*) | Approved drug | Diabetic complication |
| ErbB4  (*ERBB4*) | 2R4B | ATP binding site | Dacomitinib | Approved drug | Non-small-cell lung cancer |
|  |  |  | GW7* | Inhibitor |  |
| SHP2  (*PTPN11*) | 4RDD | Inhibitor binding site | 3LU* | Inhibitor |  |
| FGFR1 | 4WUN | ATP binding site | Intedanib | Approved drug | Colorectal cancer |
|  |  |  | 66T* | Inhibitor |  |
| ERα  (*ESR1*) | 3ERT | Ligand binding domain | 4-hydroxytamoxifen (OHT*) | Active metabolite of tamoxifen (Approved drug) | Breast cancer |
| Aromatase  (*CYP19A1*) | 3S7S | Substrate binding site | Exemestane (EXM*) | Approved drug | Breast cancer |
|  |  |  | Testosterone | Substrate |  |

*PDB ID of the protein crystal structures and ligands
