# Supplementary material for: Identification of the anti-breast cancer targets of triterpenoids in Liquidambaris Fructus and the hints for its traditional applications
Source: BMC Complement Med Ther. 2020 Nov 27;20:369. doi: 10.1186/s12906-020-03143-8 (PMC7694930; doi:10.1186/s12906-020-03143-8)
Supplement: Supplementary file 3 — Additional file 3. The NMR spectrum of LF03, LF04, LF05, LF08, LF09 and LF10. [file 12906_2020_3143_MOESM3_ESM.docx]

**Additional Fig. 2(a)** ^1^H NMR spectrum of **LF03**.

**Additional Fig. 2(b)** ^13^C NMR spectrum of **LF03**.

**Additional Fig. 2(c)** ^1^H NMR spectrum of **LF04**.

**Additional Fig. 2(d)** ^13^C NMR spectrum of **LF04**.

**Additional Fig. 2(e)** ^1^H NMR spectrum of **LF05**.

**Additional Fig. 2(f)** ^13^C NMR spectrum of **LF05**.

**Additional Fig. 2(g)** ^1^H NMR spectrum of **LF08**.

**Additional Fig. 2(h)** ^13^C NMR spectrum of **LF08**.

**Additional Fig. 2(i)** ^1^H NMR spectrum of **LF09**.

**Additional Fig. 2(j)** ^13^C NMR spectrum of **LF09**.

**Additional Fig. 2(k)** ^1^H NMR spectrum of **LF10**.

**Additional Fig. 2(l)** ^13^C NMR spectrum of **LF10**.
