# Supplementary material for: Identification of the anti-breast cancer targets of triterpenoids in Liquidambaris Fructus and the hints for its traditional applications
Source: BMC Complement Med Ther. 2020 Nov 27;20:369. doi: 10.1186/s12906-020-03143-8 (PMC7694930; doi:10.1186/s12906-020-03143-8)
Supplement: Supplementary file 6 — Additional file 6. Protein families that the molecular docking targets belonged to. [file 12906_2020_3143_MOESM6_ESM.docx]

**Additional Table 2** Protein families that the molecular docking targets belonged to

| **Superfamily** | **Family** | **Subfamily** | **Protein target** |
| --- | --- | --- | --- |
| protein kinase superfamily | protein tyrosine kinase family | epidermal growth factor receptor (EGFR) subfamily | EGFR,  ErbB4 (*ERBB4*) |
|  |  | SRC subfamily | Lck (*LCK*),  Src (*SRC*) |
|  |  | fibroblast growth factor receptor subfamily | FGFR1 |
|  |  | insulin receptor subfamily | IGF1R |
|  |  | CSF-1/PDGF receptor subfamily | VEGFR2 (*KDR*) |
|  |  |  | c-Met (*MET*) |
|  | CMGC serine/threonine protein kinase family | CDC2/CDKX subfamily | CDK2, CDK6 |
|  |  | MAP kinase subfamily | ERK2 (*MAPK1*), JNK1 (*MAPK8*),  p38 MAPK (*MAPK14*) |
|  |  | GSK-3 subfamily | GSK-3β (GSK3B) |
|  | PI3/PI4-kinase family |  | PI3Kγ (*PIK3CG*) |
|  | nuclear hormone receptor family | NR3 subfamily | ERα (*ESR1*), AR |
|  |  | NR1 subfamily | PPARγ (*PPARG*) |
|  | cytochrome P450 family |  | Aromatase (*CYP19A1*) |
|  | protein-tyrosine phosphatase family |  | PTP1B (*PTPN1*),  SHP2 (*PTPN11*) |
|  | peptidase C14A family |  | caspase-3 (*CASP3*) |
|  | poly (ADP-ribose) polymerase family |  | PARP1 |
|  | heat shock protein 70 family |  | HSC70 (*HSPA8*) |
|  | heat shock protein 90 family |  | HSP90α (*HSP90AA1*) |
|  | MDM2/MDM4 family |  | MDM2 |
|  | sigma family |  | HPGDS |
|  | annexin family |  | annexin A5  (*ANXA5*) |
|  | peptidase S1 family |  | Prothrombin  (*F2*) |
|  | peptidase A1 family |  | Rennin (*REN*) |
