# Supplementary material for: Identification of the anti-breast cancer targets of triterpenoids in Liquidambaris Fructus and the hints for its traditional applications
Source: BMC Complement Med Ther. 2020 Nov 27;20:369. doi: 10.1186/s12906-020-03143-8 (PMC7694930; doi:10.1186/s12906-020-03143-8)
Supplement: Supplementary file 7 — Additional file 7. Functionally grouped network view of the GO enrichment analysis result. [file 12906_2020_3143_MOESM7_ESM.docx]

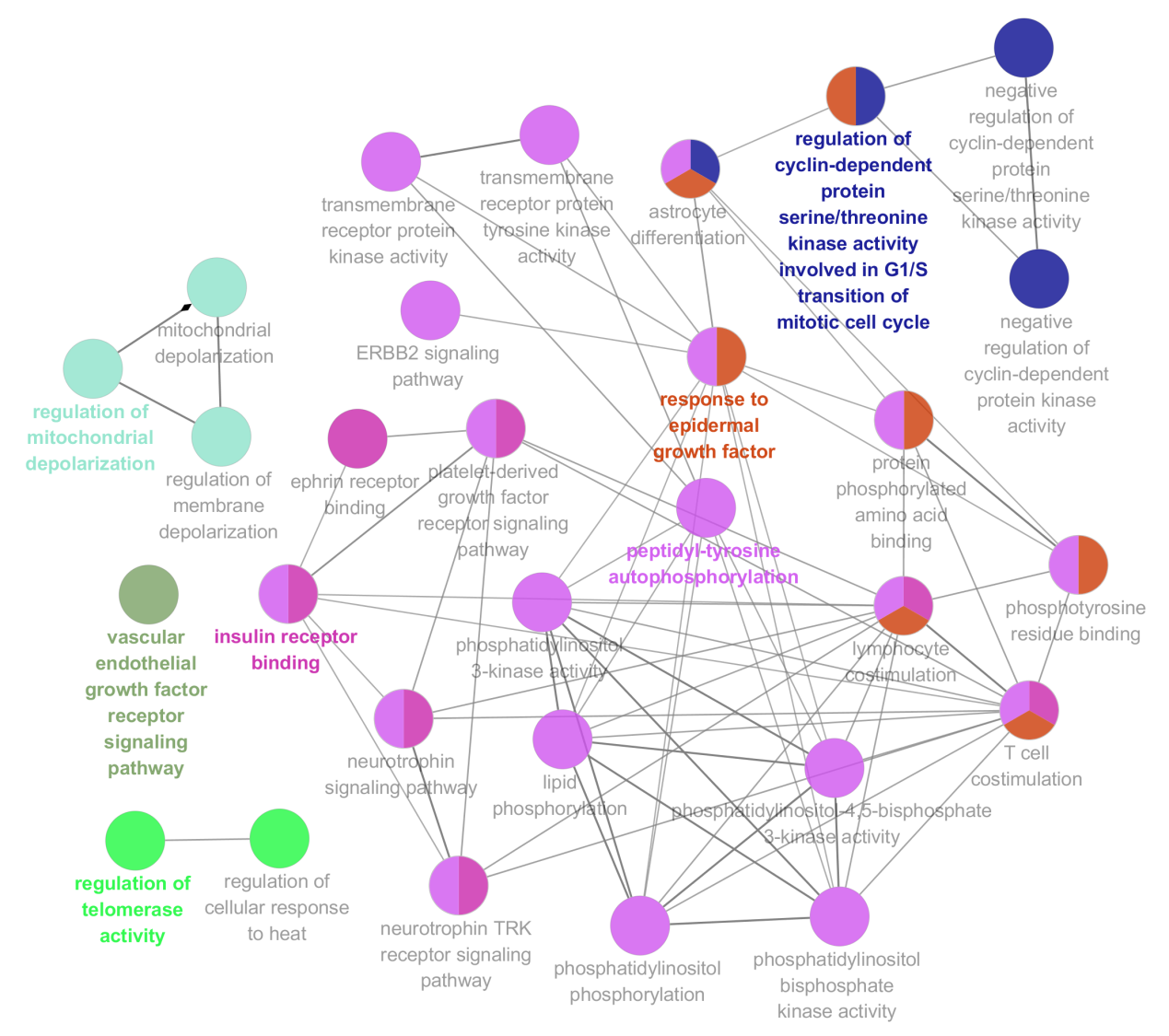


**Additional Fig. 2(a)** Functionally grouped network view of the GO enrichment analysis result. Functional groups are colored and represented by their most significant term (leading term) based on the kappa statistics. The edge thickness shows kappa-score relations between the terms.


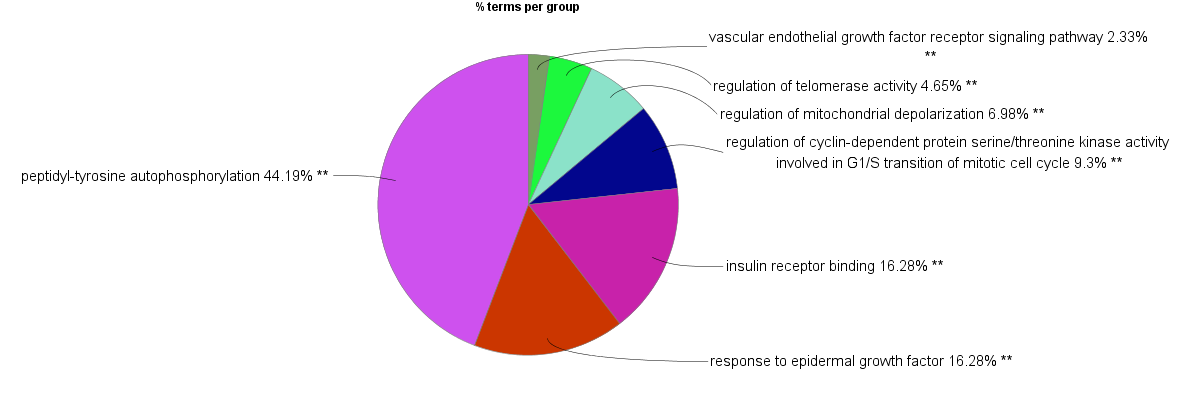


**Additional Fig. 2(b)** The proportion of functional groups in the enriched terms.
