# Supplementary figures and images for: Identification of the anti-breast cancer targets of triterpenoids in Liquidambaris Fructus and the hints for its traditional applications
Source: BMC Complement Med Ther. 2020 Nov 27;20:369. doi: 10.1186/s12906-020-03143-8 (PMC7694930; doi:10.1186/s12906-020-03143-8)

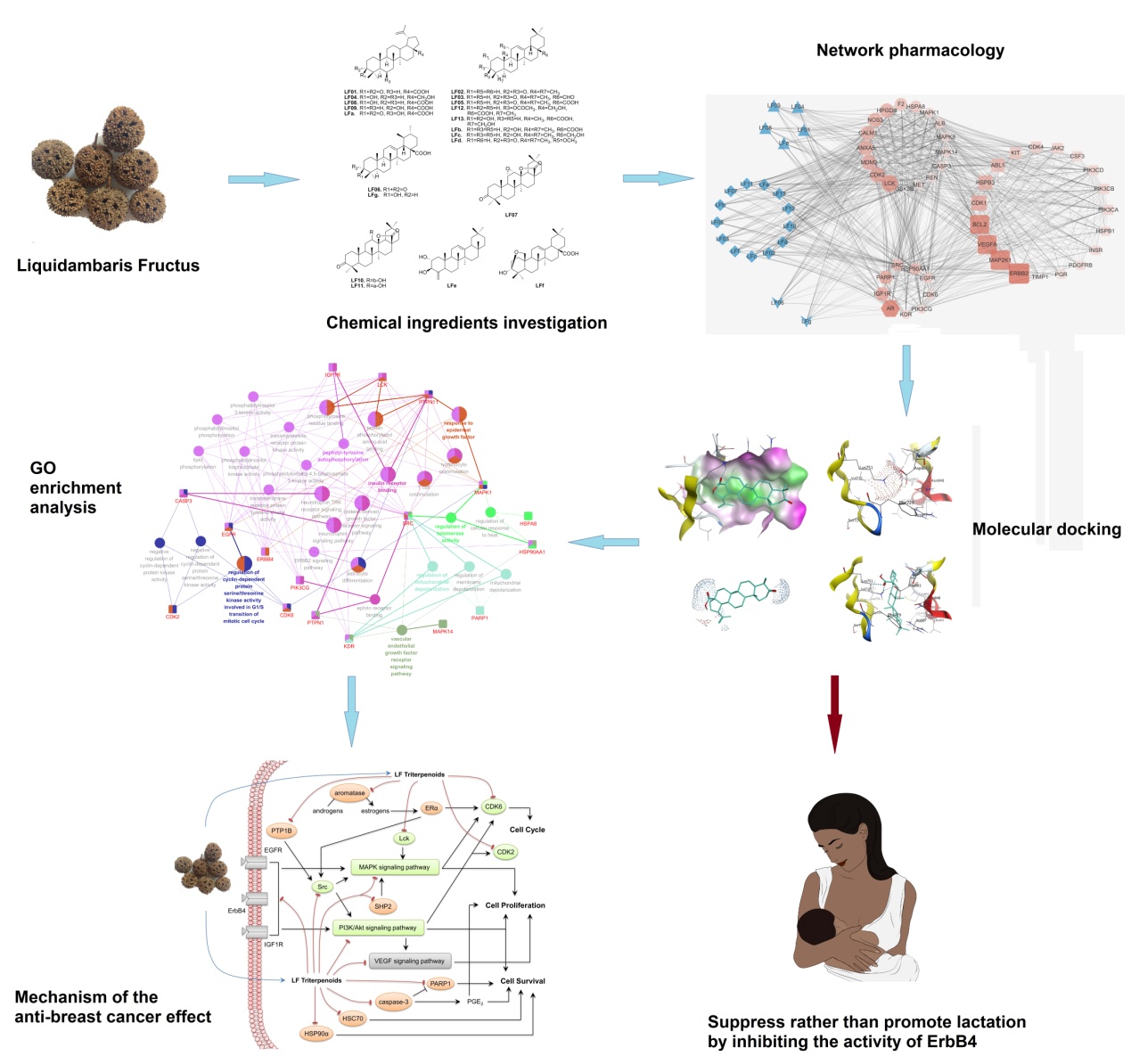


**Additional Fig. 3** The framework and research strategy of the article.

Supplement: Supplementary file 8 — Additional file 8. The framework and research strategy of the article. [file 12906_2020_3143_MOESM8_ESM.docx]
